# Isolation and Purification of Galloyl, Caffeoyl, and Hexahydroxydiphenoyl Esters of Glucoses from *Balanophora simaoensis* by High-Speed Countercurrent Chromatography and Their Antioxidant Activities In Vitro

**DOI:** 10.3390/molecules23082027

**Published:** 2018-08-14

**Authors:** Lei Fang, Tian-Tian He, Xiao Wang, Jie Zhou

**Affiliations:** 1 School of Biological Science and Technology, University of Jinan, Jinan 250022, China; fleiv@163.com (L.F.); htiantian222@163.com (T.-T.H.); 2 Shandong Key Laboratory of TCM Quality Control Technology, Shandong Analysis and Test Center, Qilu University of Technology (Shandong Academy of Sciences), Jinan 250014, China; wangx@sdas.org

**Keywords:** *Balanophora simaoensis*, glucose esters, high-speed counter-current chromatography, antioxidant activities

## Abstract

High-speed counter-current chromatography was used to separate and purify galloyl, caffeoyl, and hexahydroxydiphenoyl esters of glucoses from the aerial parts of the parasitic plant *Balanophora simaoensis* for the first time using *n*-hexane-ethyl acetate-methanol-water (1:2:1:2, *v*/*v*) as the optimum solvent system. Accordingly, 1-*O*-(*E*)-caffeoyl-3-*O*-galloyl-*β*-d-glucopyranose (**I**, 12.5 mg), 1-*O*-(*E*)-caffeoyl-3-*O*-galloyl-4,6-(*S*)-hexahydroxydiphenoyl-*β*-d-glucopyranose (**II**, 27.2 mg), and 1-*O*-(*E*)-caffeoyl-4,6-(*S*)-hexahydroxydiphenoyl-*β*-d-glucopyranose (**III**, 52.8 mg) with 98.0%, 98.5%, and 98.7% purities, respectively, were purified from 210 mg crude extract of *B. simaoensis* in a one-step separation. The structures of the glucose esters were identified by electrospray ionization mass spectrometry (ESI-MS) and nuclear magnetic resonance spectra (NMR). Their antioxidant activities were evaluated by measuring their inhibition activity on liver microsomal lipid peroxidation induced by the Fe^2+^-Cys system in vitro. Compounds **I**–**III** showed significant antioxidant activities with IC_50_ values ranging from 2.51 to 6.68 μm, respectively.

## 1. Introduction

*Balanophora simaoensis* (Balanophoraceae) indigenous to China is a parasitic plant growing on the roots of various plants and mainly distributed in the Yunnan province, China [1]. In Chinese traditional medicine, *B. simaoensis* is medicinally used as hemostatic, antidote, and antipyretic agents since ancient times [2]. Previous phytochemical investigations of *Balanophora* species revealed the presence of various types of bioactive components, such as flavanones, phenylpropanoids, lignans, steroids, triterpenes, glucose esters, and ellagitannins [3,4,5,6,7,8]. The glucose esters possessing galloyl, caffeoyl and hexahydroxydiphenoyl (HHDP) ester moieties exhibited significant antioxidant activities, thus maybe good candidates for further development with antioxidant potential [9]. In order to further study the biochemical properties of glucose esters from *B. simaoensis* and to evaluate their clinical applications, it is essential to develop an efficient method for the separation of glucose esters.

**Figure 1:**
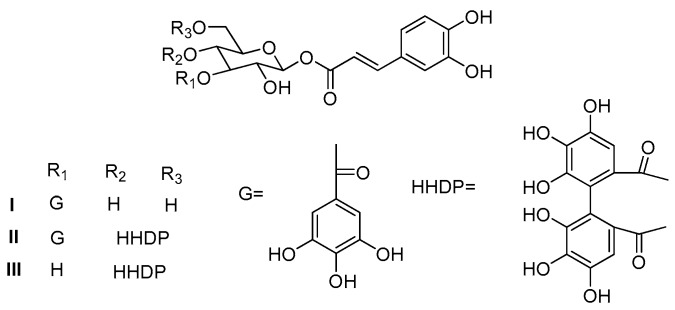
Chemical structures of three glucose esters from *B. simaoensis*.

However, conventional methods for the isolation of these glucose esters are often based on the extensive use of multiple chromatographic steps, which is time-consuming and offered low recoveries of the target products [8,10]. High-speed counter-current chromatography (HSCCC) is a liquid–liquid partition chromatography, which is capable of eliminating the solid carrier adsorption and offering total recovery of target compounds. Due to the advantages of HSCCC, it has been widely used in the separation and purification of different kinds of natural products [11,12]. To the best of our knowledge, there are few reports on the preparative separation of glucose esters from plants extracts using HSCCC to date. In the present paper, an efficient HSCCC method has been developed for isolation and purification of three glucose esters, 1-*O*-(*E*)-caffeoyl-3-*O*-galloyl-*β*-d-glucopyranose (I), 1-*O*-(*E*)-caffeoyl-3-*O*-galloyl-4,6-(*S*)-HHDP-*β*-d-glucopyranose (II), and 1-*O*-(*E*)-caffeoyl-4,6-(*S*)-HHDP-*β*-d-glucopyranose (III) from a crude sample of *B. simaoensis* (Figure 1). Furthermore, the antioxidant activities of three glucose esters were evaluated by measuring their inhibition activity on liver microsomal lipid peroxidation induced by the Fe^2+^-Cys system in vitro.

## 2. Results

### 2.1. Optimization of HPLC Conditions

**Figure 2:**
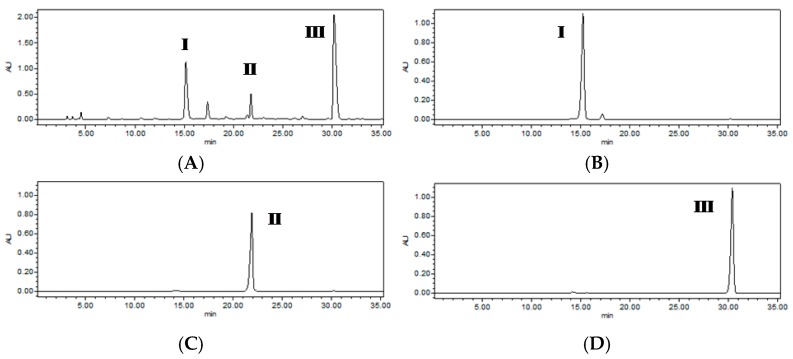
HPLC chromatogram of the crude extract from *B. simaoensis* (**A**) and the three glucose esters purified by HSCCC separation fraction (**B**–**D**).

Good results were obtained when the mobile phase was acetonitrile (solvent A) and water (solvent B) with the gradient (0 min 15% A; 35 min 70% A). The flow rate and detection wavelength were also investigated. The best results were obtained using 1.0 mL/min and 254 nm as the flow rate and detection wavelength, respectively. The crude extract of *B. simaoensis* was analyzed under the optimized HPLC conditions (Figure 2). As shown in Figure 2, peaks **I**–**III** were selected as the target for HSCCC separation in the current work.

### 2.2. Selection of the Two-Phase Solvent System

Selection of a suitable two-phase solvent system is the first and critical step in a successful separation using HSCCC. A suitable solvent system should provide ideal partition coefficient *K* values (*K*, 0.5–2) and a proper separation factor (*α* > 1.5) of the target compounds [13]. Several two-phase solvent systems were investigated and the *K* values of three glucose esters were measured in Table 1.

**Figure 3:**
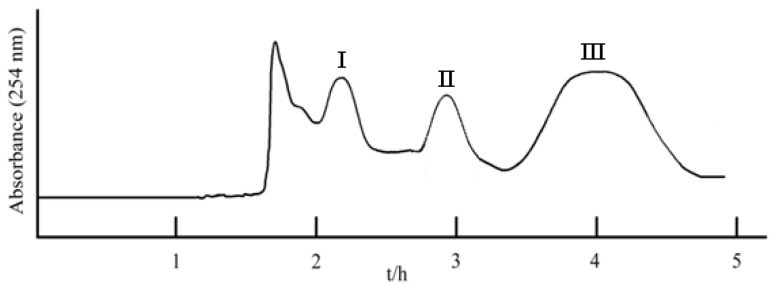
HSCCC chromatogram of the crude sample of *B. simaoensis*. Solvent system: *n*-Hexane-ethyl acetate-methanol-water (1:2:1:2, *v*/*v*); revolution speed: 850 r/min; flow rate: 2.0 mL/min; sample size: 210 mg; UV detection wavelength: 254 nm.

As shown in Table 1, the *K* values of three glucose esters were too large in the two-phase solvent systems of ethyl acetate-*n*-butanol-water (5:2:6, *v*/*v*) and ethyl acetate-ethanol-water (5:1:6, *v*/*v*), which would cause excessive band broadening and a longer elution time. Then the *n*-hexane-ethyl acetate-methanol-water (HEMWat) system was tested, which has been widely applied to separate various natural products [14]. When the solvent system changed to the HEMWat system (3:5:3:5, 2:5:2:5, 1:2:1:2), the *K* values of three glucose esters decreased. It can be seen that the *K* values in the solvent system with the volume ratio of 1:2:1:2 (*v*/*v*) were suitable for separation of the target compounds. As shown in Figure 3, good resolution and acceptable separation time could be obtained using *n*-hexane-ethyl acetate-methanol-water (1:2:1:2, *v*/*v*) as the two-phase solvent system.

### 2.3. HSCCC Separation of Three Glucose Esters

With the optimized solvent system *n*-hexane-ethyl acetate-methanol-water (1:2:1:2, *v*/*v*), the ethyl acetate extract (210 mg) of *B. simaoensis* was successfully separated and purified by HSCCC in one step. As shown in Figure 3, three glucose esters were obtained: 1-*O*-(*E*)-caffeoyl-3-*O*-galloyl-*β*-d-glucopyranose (**I**, 12.5 mg),1-*O*-(*E*)-caffeoyl-3-*O*-galloyl-4,6-(*S*)-HHDP-*β*-d-glucopyranose (**II**, 27.2 mg), and 1-*O*-(*E*)-caffeoyl-4,6-(*S*)-HHDP-*β*-d-glucopyranose (**III**, 52.8 mg). The crude extract and HSCCC fractions were analyzed by HPLC, and the HPLC chromatograms were shown in Figure 2. The purities of three glucose esters were 98.0%, 98.5%, and 98.7% purities, respectively, as determined by HPLC. The retention of the stationary phase was 71.0%, and the separation time was within 5 h in each separation run. These results demonstrated that HSCCC was a powerful and efficient tool for the preparative separation of glucose esters.

### 2.4. Identification of Separated Compounds

The structures of three glucose esters were determined by ESI-MS, ^1^H-NMR, and ^13^C-NMR analysis. The results were as follows*:*

Compound **I**: ESI-MS *m/z*: 493 [M − H]^−^. ^1^H-NMR (CD_3_OD-*d*_4_, 600 MHz) *δ*: 7.65 (1H, d, *J* = 15.0 Hz, caf-7), 7.08 (1H, d, *J* = 2.4 Hz, caf-2), 7.01 (2H, s, gal-2, 6), 6.92 (1H, dd, *J* = 2.4, 7.8 Hz, caf-6), 6.74 (1H, d, *J* = 7.8 Hz, caf-5), 6.31 (1H, d, *J* = 15.0 Hz, caf-8), 5.72 (1H, d, *J* = 7.8 Hz, glc-1), 5.24 (1H, t, *J* = 9.0 Hz, glc-3), 4.48 (1H, d, *J* = 12.6 Hz, glc-6a), 4.28 (1H, d, *J* = 12.6 Hz, glc-6b), 3.72 (1H, t, *J* = 9.0 Hz, glc-4), 3.69 (1H, t, *J* = 9.6 Hz, glc-2), 3.56 (1H, t, *J* = 9.4 Hz, glc-5).^13^C-NMR (CD_3_OD-*d*_4_, 150 MHz) *δ*: caffeoyl: 126.5 (C-1), 114.3 (C-2), 145.4 (C-3), 148.5 (C-4), 115.6 (C-5), 122.8 (C-6), 147.6 (C-7), 113.1 (C-8), 166.3 (C-9); galloyl: 119.7 (C-1), 109.5 (C-2, 6), 144.8 (C-3, 5), 138.6 (C-4), 166.5 (C-7); glucose: 94.8 (C-1), 72.5 (C-2), 79.1 (C-3), 69.8 (C-4), 78.2 (C-5), 62.1 (C-6). On comparison with the reported data [8], compound **I** was identified as 1-*O*-*(E*)-caffeoyl-3-*O*-galloyl-*β*-d-glucopyranose. 

Compound **II**: ESI-MS *m/z*: 795 [M − H]^−^. ^1^H-NMR (CD_3_OD-*d*_4_, 600 MHz) *δ*: 7.64 (1H, d, *J* = 16.0 Hz, caf-7), 7.06 (1H, d, *J* = 2.4 Hz, caf-2), 7.03 (1H, dd, *J* = 2.4, 7.8 Hz, caf-6), 7.02 (2H, s, gal-2, 6), 6.78 (1H, d, *J* = 7.8 Hz, caf-5), 6.60, 6.45 (each 1H, s, HHDP-3, 3′), 6.32 (1H, d, *J* = 16.0 Hz, caf-8), 5.76 (1H, d, *J* = 7.8 Hz, glc-1), 5.42 (1H, t, *J* = 9.6 Hz, glc-3), 5.32 (1H, dd, *J* = 6.6, 13.2 Hz, glc-6a), 5.07 (1H, t, *J* = 9.6 Hz, glc-4), 4.30 (1H, brdd, *J* = 6.6, 9.6 Hz, glc-5), 3.84 (1H, t, *J* = 8.0 Hz, glc-2), 3.84 (1H, d, *J* = 13.2 Hz, glc-6).^13^C-NMR (CD_3_OD-*d*_4_, 150 MHz) *δ*:caffeoyl:126.6 (C-1), 114.4 (C-2), 145.3 (C-3), 148.5 (C-4), 115.4 (C-5), 122.7 (C-6), 147.5 (C-7), 113.2 (C-8), 166.0 (C-9); galloyl: 119.6 (C-1), 109.1 (C-2, 6), 145.1 (C-3, 5), 138.4 (C-4), 166.3 (C-7); HHDP: 115.3, 115.0 (C-1, 1′), 125.2, 125.0 (C-2, 2′), 106.9, 107.1 (C-3, 3′), 144.4, 144.5 (C-4, 4′), 136.2, 136.5 (C-5, 5′), 143.1, 143.4 (C-6, 6′), 167.8, 168.1 (C-7, 7′); glucose: 95.1 (C-1), 71.4 (C-2), 74.8 (C-3), 70.1 (C-4), 75.2 (C-5), 65.3 (C-6).Compared with the reported data [8], compound **II** was identified as 1-*O*-*(E*)-caffeoyl-3-*O*-galloyl-4,6-(*S*)-hexahydroxydiphenoyl-*β*-d-glucopyranose.

Compound **III**: ESI-MS *m/z*: 643 [M − H]^−^. ^1^H-NMR (CD_3_OD-*d*_4_, 600 MHz) *δ*: 7.66 (1H, d, *J* = 16.0 Hz, caf-7), 7.04 (1H, d, *J* = 2.4 Hz, caf-2), 6.98 (1H, dd, *J* = 2.4, 8.0 Hz, caf-6), 6.79 (1H, d, *J* = 8.0 Hz, caf-5), 6.59, 6.48 (each 1H, s, HHDP-3, 3′), 6.30 (1H, d, *J* = 16.0 Hz, caf-8), 5.69 (1H, d, *J* = 8.0 Hz, glc-1), 5.30 (1H, dd, *J* = 6.0, 12.6 Hz, glc-6), 4.86 (1H, t, *J* = 9.6 Hz, glc-4), 4.13 (1H, brdd, *J* = 6.0, 9.6 Hz, glc-5), 3.80 (1H, d, *J* = 12.6 Hz, glc-6), 3.79 (1H, t, *J* = 9.6 Hz, glc-3), 3.56 (1H, t, *J* = 10.0 Hz, glc-2). ^13^C-NMR (CD_3_OD-*d*_4_, 150 MHz) *δ*: caffeoyl: 127.7 (C-1), 115.5 (C-2), 146.3 (C-3), 149.5 (C-4), 116.4 (C-5), 123.2 (C-6), 148.5 (C-7), 114.2 (C-8), 166.5 (C-9); HHDP: 115.0, 115.3 (C-1, 1′), 125.3, 125.5 (C-2, 2′), 106.4, 107.2 (C-3, 3′), 144.4, 144.8 (C-4, 4′), 136.3, 136.5 (C-5, 5′), 143.7, 143.8 (C-6, 6′), 167.8, 168.1 (C-7, 7′); glucose: 96.1 (C-1), 72.7 (C-2), 74.5 (C-3), 73.1 (C-4), 75.5 (C-5), 64.7 (C-6). According to literature [8], compound **III** was identified as1-*O*-*(E*)-caffeoyl-4,6-(*S*)-hexahydroxydiphenoyl-*β*-d-glucopyranose.

### 2.5. Antioxidant Activities of Three Glucose Esters

The antioxidant activities of three glucose esters were evaluated by measuring their inhibition activity on liver microsomal lipid peroxidation induced by the Fe^2+^-Cys system in vitro. As shown in Table 2, compounds **I**–**III** showed significant antioxidant activities with IC_50_ values ranging from 2.51 to 6.68 μm, respectively.

## 3. Materials and Methods 

### 3.1. Apparatus

The HSCCC instrument employed in the study was a model TBE-300C high-speed countercurrent chromatography apparatus (Tauto Biotech, Shanghai, China) with three multilayer coils connected in series (total volume: 315 mL; I.D.: 1.6 mm), which was equipped with a model TBP-5002S constant-flow pump (Tauto Biotech, Shanghai, China), a Model TBD2000 UV detector (Tauto Biotech, Shanghai, China) operating at 254 nm, and a model HW2000 workstation (Tauto Biotech, Shanghai, China). The temperature of the HSCCC column was controlled at 25 °C using a Model DC-0506 circulatory temperature regulator (Tauto Biotech, Shanghai, China).

The analytical HPLC system consisted of a Waters Empower system (Milford, MA, USA) with a model 600 pump, a model 600 multi-solvent delivery system, a model 996 diode-array detector, and an Empower workstation. The ESI-MS data were obtained by an Agilent 1100/MSG1946 mass spectrometer (Agilent, Santa Clara, CA, USA). The ^1^H- and ^13^C-NMR experiments wereperformed on a Varian 600 MHz NMR spectrometer (Varian, Palo Alto, CA, USA).

### 3.2. Reagents and Materials

The solvents used for the HSCCC separation and antioxidant assay were analytical grade, and were purchased from Tianjing Chemical Factory (Tianjing, China). Acetontrile was HPLC grade and purchased from Siyou Special Reagent Factory (Tianjin, China). The PBS, liver microsomes, and cysteine were bought from Beijing Solarbio Science & Technology Co., Ltd. (Beijing, China). The FeSO_4_ and thiobarbituric acid were obtained from Sigma (St. Louis, MO, USA). The whole plants of *B. simaoensis* were collected in Yunan, China, and identified by Dr. Jia Li (College of Pharmacy, Shandong University of Traditional Chinese Medicine).

### 3.3. Preparation of Crude Sample

The whole plants of *B. simaoensis* (1.5 kg) were powdered and extracted with MeOH at room temperature. The extract was filtered and concentrated in vacuo to afford the crude extract (210 g). Then, the extract was suspended in water (500 mL) and extracted by *n*-hexane (3 × 500 mL), ethyl acetate (3 × 500 mL), and *n*-butanol (3 × 500 mL) in sequence. The ethyl acetate extract were evaporated to dryness, yielding 42 g of ethyl acetate extract for further HSCCC separation.

### 3.4. Selection of Two-Phase Solvent System

The selection of two-phase solvent system was based on the partition coefficient (*K*) of the three target compounds. The *K* values were determined by HPLC [15]; approximately 2 mg of crude extract was added to a test tube, and 2 mL of equilibrated two-phase solvent system was added. The test tube was shaken violently for several minutes to make the sample fully dissolve. Then, an equal volume of the organic and aqueous phases were analyzed by HPLC. The *K* value was expressed as the peak area of the compound in the upper phase divided by that in the lower phase.

### 3.5. HSCCC Separation

In the present study, the two-phase solvent system of *n*-hexane-ethyl acetate-methanol-water (1:2:1:2, *v*/*v*) was used for the HSCCC separation. The solvent system was thoroughly equilibrated in a separation funnel by repeatedly vigorously shaking at room temperature and separated shortly prior to use. The sample solution was prepared by dissolving the dried extract in the mixture solution of lower phase and upper phase (1:1, *v*/*v*) of the solvent system.

The upper phase was firstly pumped to the multiplayer coiled column as the stationary phase. Then, the lower aqueous phase was pumped into the column at a suitable flowrate of 2 mL/min, while the apparatus was rotated at a speed of 850 rpm. After hydrodynamic equilibrium was established, the sample solution was injected into the HSCCC and separated with a head-to-tail mode. The effluent of the HSCCC was continuously monitored by a UV detector at 254 nm. The peaks were collected manually according to the elution profile and then analyzed by HPLC. Finally, the retention of the stationary phase was obtained by measuring the ratio of the organic phase to the whole volume in the multilayer-coiled columns.

### 3.6. HPLC Analysis of HSCCC Fractions

The peak fractions from the crude sample and HSCCC separation were analyzed by HPLC equipped with a Inertsil-ODS-SP column (250 × 4.6 mm, 5 μm, GL Sciences Inc., Tokyo, Japan) at room temperature. The mobile phase of acetonitrile (A) and water (B), with gradient 0 min 15% A; 35 min 70% A, was applied at a flow rate of 1.0 mL/min. Chroma grams were recorded at 254 nm. 

### 3.7. Identification of HSCCC Fractions

The fractions of the target compounds obtained from the HSCCC were determined by MS, ^1^H- and ^13^C- NMR spectra. ESI-MS spectra were analyzed by an Agilent 1100/MS-G1946 (Agilent, Santa Clara, CA, USA) mass spectrometer in the negative ionzation mode. The ^1^H- and ^13^C-NMR spectra were obtained on a Varian-600NMR spectrometer (Varian, Palo Alto, CA, USA).

### 3.8. In Vitro Antioxidant

The antioxidant activities of compounds **I**–**III** were evaluated by measuring their inhibition activities on liver microsomal lipid peroxidation induced by the Fe^2+^-Cys system in vitro [16]. Vitamin E was used as a positive control. Briefly, 1 mg/mL microsomal protein, different concentrations of the test compound or vehicle and 0.2 μm cysteine in 0.1 M PBS (pH 7.4) were incubated for 15 min at 37 °C, 0.5 μm FeSO_4_ was added, mixed, and incubated for 15 min at 37 °C again. The reaction was terminated by addition an equal volume of 20% TFA. The mixture was centrifuged at 3000× *g* rpm for 10 min. The supernatant (1 mL) was reacted with 0.67% (*w*/*v*) thiobarbituric acid in a boiling H_2_O bath for 10 min. After cooling, the absorbance was read at 532 nm, and the inhibitory rate was calculated. The value of IC_50_ was calculated by Origin 8.0 Version software (OriginLab, Northampton, MA, USA) from the graph plotting inhibition percentage.

## 4. Conclusions

In this study, an efficient HSCCC method was developed and successfully applied to the separation and purification of glucose esters from the crude extract of *B. simaoensis*. From 210 mg of the crude sample, three glucose esters with purity all over 98.0% were preparative separated and showed significant antioxidant activities by measuring their inhibition activity on liver microsomal lipid peroxidation induced by the Fe^2+^-Cys system in vitro. The study is of great reference value for preparative separation of glucose esters with high purity from the *Balanophora* plants, which also demonstrated that HSCCC is a powerful protocol for quick and efficient separation and purification of active compounds from natural products.

## Figures and Tables

**Table 1:** The *K* values of the target compounds in several solvent system.

Solvent System (*v*/*v*)	*K*		
	I	II	III
ethyl acetate-*n*-butanol-water (5:2:6)	9.72	15.20	18.83
ethyl acetate-ethanol-water (5:1:6)	4.75	11.46	19.11
*n*-hexane-ethyl acetate-methanol-water (3:5:3:5)	0.28	0.59	1.82
*n*-hexane-ethyl acetate-methanol-water (2:5:2:5)	0.89	1.71	3.28
*n*-hexane-ethyl acetate-methanol-water (1:2:1:2)	0.57	1.02	2.13

**Table 2:** Antioxidant activities of Compounds **I**–**III**.

Compounds	IC_50_Values (μm)
**I**	6.68
**II**	2.51
**III**	3.74
Vitamin E	2.08
